# Report of a Case of Diphtheria

**Published:** 1888-10

**Authors:** 


### REPORT OF A CASE OF DIPHTHERIA.

Mr. President and Gentlemen of the Hahnemannian Society: The case I have to report is one of a boy five (5) years old, of very dark complexion, with black hair and eyes, previously of good health, and with a naturally strong constitution. I first saw him (Sunday) June 24th last. He had been "dumpish," his father said, for two days; wanted to lie down. An examination showed considerable swelling of the tonsils, some redness of the fauces, and slight appearance of diphtheritic membrane. The child would not answer questions, and as both tonsils seemed equally affected, with *no* characteristic indications, I concluded to allow the case to develop, and gave placebos. Knowing I should be out of town on the following day, I directed the father of the child to consult my neighbor, Dr. Julius Schmitt. He did so, and Dr. Schmitt, thinking I had exhibited a remedy already, continued Sac. lactis. On Tuesday, the 26th, was called to see the child; found him with considerable fever; pulse, 114; severe pain on swallowing, and an extensive exudation nearly covering both tonsils, which were much swollen, though the left one was the worst. The membrane had a dirty yellow color; could get no subjective symptoms. Prescribed Lachesis$^{cm}$, one dose dry on tongue, with Sac. lactis every hour in water.

June 27th.—Found no improvement in the case, pulse is 122; respiration somewhat impeded, and a croupy cough, which evidently hurts the patient, has appeared. Much pain on swallowing; is quite restless; no thirst. Lach.$^{cm}$, one dose, and Sac. lac.

June 28th.—Continuation of same symptoms, though all are more severe, and a decided increase of the croupy symptoms. Pulse, 128. Considerable yellow, stringy mucus discharged from fauces; tongue coated yellowish white, and is hard and small. Prescribed Kali bich.$^{cm}$, one dose, and Sac. lactis.

June 29th.—Child is worse; a little bloody discharge from the nose, which smells horribly and makes the nose and upper lip sore; pulse, 130; croupy cough is more severe and makes the patient cry.

June 30th.—Respiration very croupy; sawing respiration; expiration more difficult than inspiration; increase of bloody pus from the nostrils; is very restless. Prescribed Aconite$^{2c}$ (Dunham), one dose.

July 1st.—Was called out of bed by father of child, who reported him much worse, said they hardly expected him to live until morning. Feeling much discouraged, I consulted Dr. Schmitt, and while awaiting him looked at Hering's *Guiding Symptoms.* Under Lac caninum I found the requirements of the case, a perfect picture appearing there under the Throat Symptoms. On seeing the child I found he had had several severe hemorrhages from the mouth and nose. The odor was still worse, being almost more than one could bear. Bloody pus was discharging from the nostrils so corrosive as to produce desquamation of the skin of the upper lip. Very great restlessness, child throwing himself about the bed in his distress; pulse, 168. It required very great effort to carry on respiration; a great deal of croupy cough which caused much suffering, the boy crying nearly all of the time. Tonsils swollen until they touched, and covered with a very thick membrane; uvula nearly covered, and where clear, of a bright redness, not markedly shining. Of course, I gave a very unfavorable prognosis. Also Lac caninum$^{cm}$, one dose dry on tongue. At half-past eight P. M. saw him again; was much more quiet, his father saying he has slept some nearly all day. Improvement set in one hour after taking the medicine; discharge from nose and mouth less. Pulse, 144, being a fall of twenty-four beats in twelve hours. Since six o'clock P. M. restlessness has been increasing. Still cries when coughing. Gave another powder of Lac caninum$^{cm}$, followed by Sac. lactis.

July 2d, ten A. M.—Found the boy sleeping and breathing easily; no discharge from nostrils; some shreds of membrane coming from the mouth; pulse, 114. Gave Lac caninum$^{cm}$, twelve powders, directing that he should receive one every twelve hours. He seemed to need a repetition of the dose at the end of that time.

July 3d.—Child is bright and playing; no cough; swelling of fauces much decreased; pulse, 96. His convalescence from this time was very rapid. He received no other remedy and had no sequelæ whatever. Lest there might be some question as to the diagnosis of this case, I will add that the child's father had only just recovered from a severe attack of diphtheria, and since there have been two other cases in the same family. The other cases all three yielding readily to Lachesis$^{cm}$.

The case just reported has been of great interest to me, as a little more than two years ago I lost a case with identical symptoms. I wish to ask if the intensely corrosive discharge which accompanied this case may not be an indication for Lac caninum.

Dr. Carr—It is a question with me whether you ought to have repeated the dose once in twelve hours.

Dr. Brownell—He was always worse about that interval, and one dose of the remedy would always quiet him.

Dr. Carr—Did you try a powder of Sac. lac.?

Dr. Brownell—No, I do not think the child was old enough to know the difference. As to the cause of the disease, I believe that it was from some garbage, brought from the city by the Hookers Nurseries.

Dr. Schmitt—This case shows plainly the force of the pure dynamic remedy; this case was no coincidence.

Dr. Baker—Section 160 of the *Organon* reads: "The dose of a homœopathic remedy can scarcely be reduced to such a degree of minuteness as to make it powerless to overcome, and completely cure an analogous, natural disease of recent origin, and undisturbed by injudicious treatment."

Dr. Schmitt—Hahnemann would surely think so now if he knew we used the MM potencies.

Moved and seconded the paper be accepted and published in THE HOMŒOPATHIC PHYSICIAN. Carried.

Dr. Carr was appointed essayist for the next meeting. Adjourned to Dr. Carr's office in one month.

W. H. BAKER, M. D., *Secretary*.

